# Single-cell sequencing to multi-omics: technologies and applications

**DOI:** 10.1186/s40364-024-00643-4

**Published:** 2024-09-27

**Authors:** Xiangyu Wu, Xin Yang, Yunhan Dai, Zihan Zhao, Junmeng Zhu, Hongqian Guo, Rong Yang

**Affiliations:** 1 Department of Urology, Nanjing Drum Tower Hospital, Affiliated Hospital of Medical School, Nanjing University, 321 Zhongshan Road, Nanjing, 210008 Jiangsu China; 2 Medical School, Nanjing University, Nanjing, China; 3 Department of Oncology, Nanjing Drum Tower Hospital, Affiliated Hospital of Medical School, Nanjing University, Nanjing, China

**Keywords:** Single-cell multi-omics, scRNA-seq, scTCR-seq, scBCR-seq, Spatial transcriptomics, Proteomics, Microbiome, Metabolome, Computational biology

## Abstract

Cells, as the fundamental units of life, contain multidimensional spatiotemporal information. Single-cell RNA sequencing (scRNA-seq) is revolutionizing biomedical science by analyzing cellular state and intercellular heterogeneity. Undoubtedly, single-cell transcriptomics has emerged as one of the most vibrant research fields today. With the optimization and innovation of single-cell sequencing technologies, the intricate multidimensional details concealed within cells are gradually unveiled. The combination of scRNA-seq and other multi-omics is at the forefront of the single-cell field. This involves simultaneously measuring various omics data within individual cells, expanding our understanding across a broader spectrum of dimensions. Single-cell multi-omics precisely captures the multidimensional aspects of single-cell transcriptomes, immune repertoire, spatial information, temporal information, epitopes, and other omics in diverse spatiotemporal contexts. In addition to depicting the cell atlas of normal or diseased tissues, it also provides a cornerstone for studying cell differentiation and development patterns, disease heterogeneity, drug resistance mechanisms, and treatment strategies. Herein, we review traditional single-cell sequencing technologies and outline the latest advancements in single-cell multi-omics. We summarize the current status and challenges of applying single-cell multi-omics technologies to biological research and clinical applications. Finally, we discuss the limitations and challenges of single-cell multi-omics and potential strategies to address them.

## Introduction

Transcriptomic analysis has provided fundamental insights into the study of gene expression in exploring the functions related to the process of life development, disease progression, and drug action, etc. Over the past decade, bulk RNA sequencing has shed light on biological functions from a pooled cell population transcriptomic perspective. However, it represents an average across the myriad of cells within a tissue, merely reflecting the characteristics of cell populations or perhaps predominantly the information of the most numerous cells. Moreover, bulk RNA sequencing neither elucidates the variations of a sample at the single-cell level nor reflects the expression levels of rare cells.

To tackle these constraints, scRNA-seq has revolutionized biomedical science by single-cell expression profiling. This technology enables detailed exploration of genetic information at the cellular level across various tissues and diseases, capturing the inherent heterogeneity within samples. Since Tang et al. pioneered sequencing technology on a single cell in 2009 [1], the methodology has undergone continuous refinement and maturation. These advancements have facilitated the development of full-length transcriptome profiling, high-throughput capabilities, and high-sensitivity scRNA-seq [2–4].

**Fig. 1:**
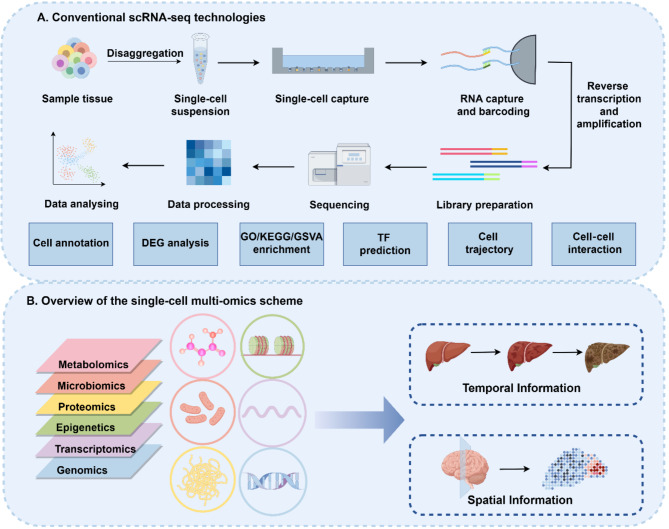
(**A**) Conventional scRNA-seq technologies. (**B**) Overview of the single-cell multi-omics scheme

Although well-established scRNA-seq has achieved great success and wide applications in the research field, it has also triggered new thinking because of its limitations. Cellular information extends well beyond RNA sequencing, encompassing the genome, epigenome, proteome, metabolome, etc., along with crucial details about spatial relationships and dynamic alterations (Fig. 1B). Therefore, scientists continually explore new methods for single-cell analysis, providing technical support to unveil the secrets of cells.

Single-cell multi-omics technologies have emerged. They refer to the simultaneous measurement of various types of data in the same cell, allowing for an accurate and detailed depiction of the cellular state. Integrating single-cell transcriptomic sequencing with comprehensive multi-omics data to map their inherent connections represents a critical and inevitable trend toward a more nuanced, multidimensional understanding of life development and the mechanisms underlying diseases.

These cutting-edge methods break through the limitations of the conventional scRNA-seq, offering an exciting solution to explore how cellular modalities affect cell state and function. Single-cell T cell receptor sequencing (scTCR-seq) [5] and single-cell B cell receptor sequencing (scBCR-seq) [6] effectively delineate the repertoires of T and B cells, respectively, which can reveal the immune system state. Integration with single-cell proteomics (CITE-seq) enriches the information with proteomics that is both similarities and discrepancies with the transcriptome [7]. Coupled with single-cell assay for transposase-accessible chromatin using sequencing (scATAC-seq), researchers gain insights into chromatin accessibility, identifying active regulatory sequences and potential transcription factors (TFs). Besides, deciphering temporal and spatial information at the single-cell level is fundamental for biological research. Although most temporal data is inferred via computational biology technology or scRNA-seq atlas created at multiple time points, the experimental method to unveil newly synthesized RNA is another way [8]. Spatial transcriptomics technologies merge tissue sectioning with single-cell sequencing to compensate for the inability of scRNA-seq to characterize spatial locations. It is worth mentioning that computational biology is necessary for integrating and analyzing single-cell multi-omics data. The information content varies across different modalities. How to integrate and process these data is of utmost importance. The standard workflow to analyze the multimodal datasets is necessary for a broadly applicable strategy of single-cell multi-omics. Additionally, computational biology methods are already widely employed to simulate related different dimension information.

The holistic view created by single-cell multi-omics technologies is crucial for understanding the complexities of biology, providing insights into cellular diversity, disease mechanisms, and potential therapeutic targets. In 2019, single-cell multimodal omics was selected as Method of the Year [9]. In this review, we encapsulate the advancements and applications of traditional scRNA-seq, outline various single-cell multi-omics methodologies, and explore their biological and clinical implications, while also contemplating current limitations and future directions.

## Conventional single-cell sequencing

scRNA-seq, stepping onto the stage of history, has completely reshaped the study approach of the complexity and heterogeneity within individual cells. scRNA-seq technologies mainly involve microfluidic chips, microdroplets, and microwell-based approaches, which have been well-introduced and compared in previous articles [10, 11]. The main experimental steps of scRNA-seq encompass preparing single-cell suspension, isolating individual cells, capturing their mRNA, conducting reverse transcription and nucleic acid amplification, and building a transcriptome library (Fig. 1A).

Analysis of scRNA-seq via bioinformatics is another cornerstone for visualizing and understanding the underlying patterns and insights within the data. Tools for analyzing scRNA-seq data are written in a variety of programming languages, with R and Python being the most prominent. R-based representative software includes Seurat, SingleCellExperiment, and SingleR, while Python-based representative software includes Scanpy, Loom, and AnnData. Data preprocessing involves implementing data quality control, aligning sequences to reference genomes, and generating expression matrices. Subsequent analyses typically utilize formats like Seurat, SingleCellExperiment, AnnData, or Loom. The general analysis workflow includes (1) filtering data based on doublets, mitochondrial content, erythrocytes, etc., (2) selecting features as highly variable genes, and (3) dimension reduction, including principal component analysis (PCA), uniform manifold approximation and projection (UMAP), or t-distributed stochastic neighbor embedding (t-SNE). The advanced analyses aim to answer the biological questions. Clustering and annotation of cell types answer what kinds of cell types are there. At the gene level, differentially expressed genes (DEGs) and gene enrichment, which includes gene ontology (GO), kyoto encyclopedia of genes and genomes (KEGG), and gene set variation analysis (GSVA), aim to identify the differential genes between cell types or specimens, as well as to clarify their associated pathway profiles. Inferring TFs by single-cell regulatory network inference and clustering (SCENIC) provides the essential gene regulatory network [12]. At the cell level, cell-cell communication offers speculation from the perspective of cellular interactions [13] while cell trajectory analysis incorporates the temporal information (Fig. 1A) [14]. Furthermore, inferring copy number variations (CNVs) with inferCNV analysis by comparing gene expression levels in cells with those in a reference genome is particularly crucial in cancer biology [15].

High cost and batch effects remain the major obstacles for large cohort studies on scRNA-seq. To overcome these constraints, the integration of multiple samples for large-scale scRNA-seq analysis has become a prevalent practice in research. Batch effects, hampering data integration, may arise from different experimental conditions, such as varying chips, sequencing lanes, or timing of cell processing. Integrating data from multiple experiments requires the use of employ algorithms such as Seurat’s canonical correlation analysis (CCA), mutual nearest neighbors (MNN), or Harmony to batch correction [16, 17].

**Fig. 2:**
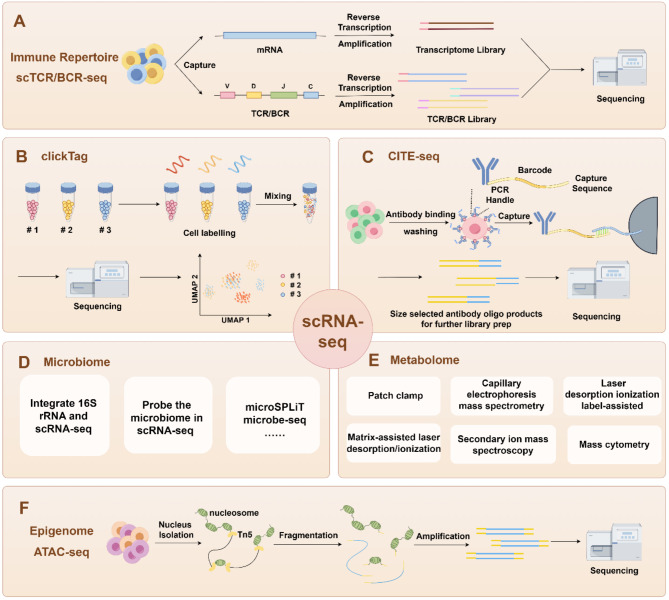
A brief method and principle of single-cell multi-omics technologies for (**A**) scTCR/BCR-seq, (**B**) ClickTag, (**C**) proteome, (**D**) microbiome, (**E**) metabolome, (**F**) epigenome

Sample multiplexed scRNA-seq is another solution, establishing an efficient method for massively parallel species-mixing experiments [18–22]. Currently, the predominant approach for this technology involves tagging individual samples with DNA oligonucleotides (oligos) barcodes before pooling them together, including lipid-tagged DNA [21], chemical cross-linking reaction [19], and genetic barcodes [20]. This technology is pragmatic, multiplexing via DNA oligos and demultiplexing conducted via bioinformatics independently of genetic background and sample origin, and is compatible with other omics technologies, ensuring that the unique biological characteristics of individual samples are maintained. Consequently, it inherently avoids batch effects or the loss of biological characteristics post-debatching. Recently, Zhao et al. reported an improved ClickTags method to enable the use in live-cell samples empowered by “click chemistry”, and eliminated the requirement for methanol fixation of samples (Fig. 2B). Moreover, this method has been successfully utilized across various murine cells and human samples of bladder cancer that have undergone freeze-thaw cycles, demonstrating its applicability to diverse single-cell specimens [22].

## New advances in single-cell sequencing

Although high-throughput single-cell sequencing has revealed the gene expression characteristics within the majority of physiological and diseased cells, the complexity of cells extends far beyond its scope. There is still a need to understand and decode the secrets of cells from multiple dimensions. With the thriving development of biology, chemistry, bioinformatics, and other advanced technologies, researchers are continuously developing new technologies and methods for single-cell sequencing. Here, we summarize new advances in single-cell sequencing, which can be major trends in future development.

### Temporal information

**Fig. 3:**
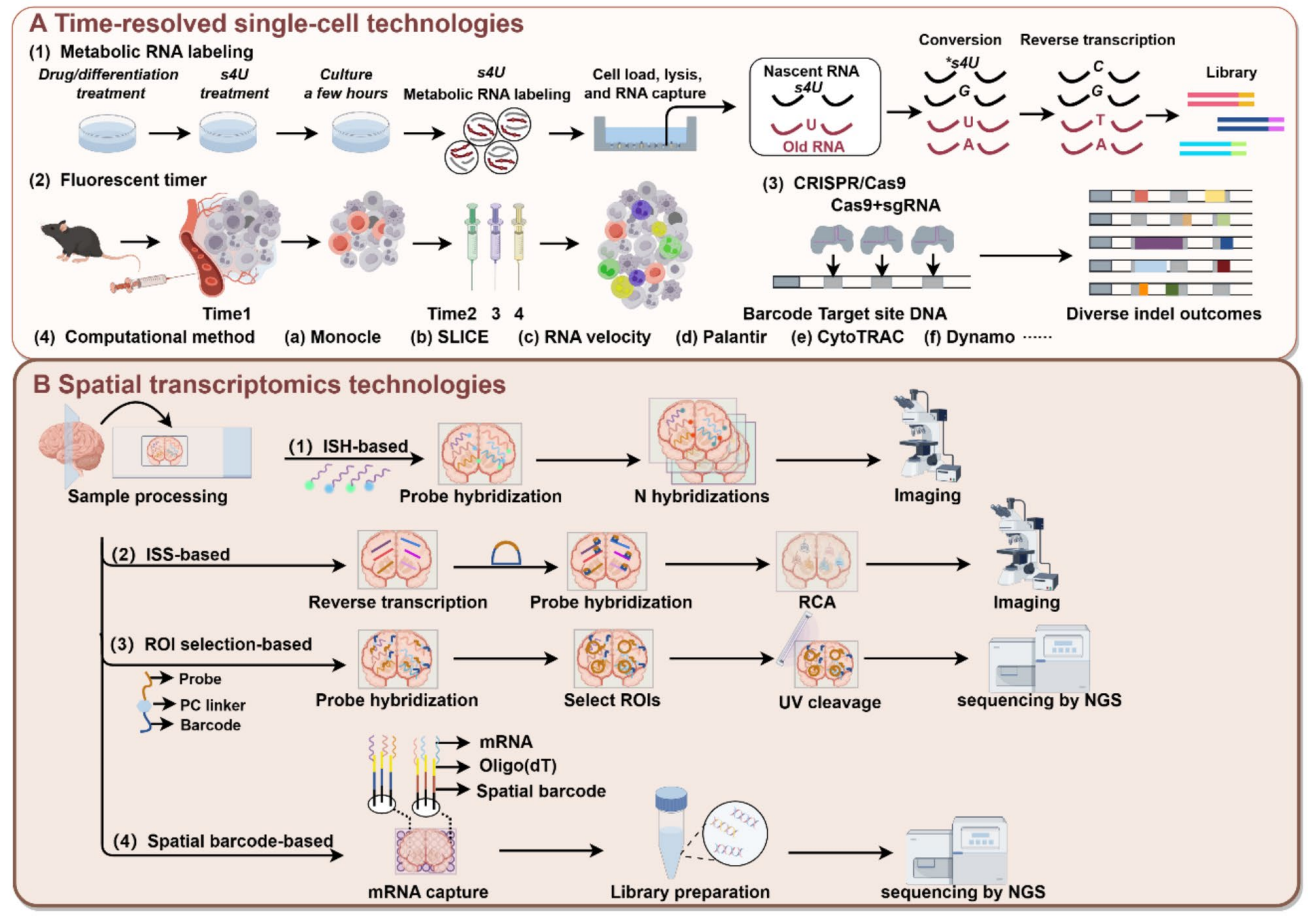
The principles of key technologies in the dimensions of (**A**) time and (**B**) space at the single-cell level

One major limitation of current scRNA-seq approaches is that they only provide static RNA expression profiles. In reality, cells are constantly undergoing dynamic changes, whether during development and differentiation, disease progression, and pre- and post-treatment, etc. Frequently, scRNA-seq is conducted at various time points to gain valuable insights into the development or response process [23–25]. Time-resolved scRNA-seq is primarily studied by experimental approaches or computational tools, allowing for the inference or acquisition of dynamic data (Fig. 3A). This enables the study of time-resolved scRNA-seq.

### Computational method

Computational tools can endow scRNA-seq data, which capture only a static snapshot at a time, with inferred temporal information without resorting to any experimental technologies. These approaches are commonly referred to as pseudotime analysis. Pseudotime analysis, also known as trajectory inference, ranks potential dynamic processes in cells based on the heterogeneity of transcriptional expression levels. These approaches effectively combining computational and biological methods, have gained wide acceptance and popularity, and have become a common advanced tool in scRNA-seq analysis. The structure of dynamic processes can be linear, nonlinear, or branching. Commonly used software includes Monocle [14], RNA velocity [26], Palantir [27], CytoTRACE [28], and others.

Monocle is an unsupervised algorithm designed for pseudotime inference analysis, capitalizing on the high variability in gene expression levels. The latest version, Monocle3, utilizes UMAP for trajectory inference [14]. Entropy-based pseudotime analysis is also a method. It leverages the concept that entropy is negatively correlated with cell differentiation states. Higher entropy values suggest a greater stemness, indicating a more primitive and undifferentiated state [29].

RNA velocity is a prevalent technology in scRNA-seq analysis [26]. It operates on the principle that, during dynamic regulatory processes, unspliced (nascent) mRNA always appears before spliced (mature) mRNA. By assessing the abundance of both unspliced and spliced mRNA, one can reveal indicators of dynamic changes in the transcriptome over time.

Palantir is a pseudo-time algorithm based on stochastic processes that, through dimensionality reduction and manifold analysis, effectively captures the continuity of cell states and models the transition from low-differentiation cells to terminally differentiated cells [27]. It can be applied to a variety of tissue types and is particularly well-suited for addressing less-studied differentiation systems.

CytoTRACE leverages gene counting and expression to reconstruct cell trajectories, enabling the prediction of relative differentiation states of single cells based on single-cell RNA expression data, without being constrained by specific time scales or the presence of continuous developmental processes in the data [28]. Additionally, CytoTRACE is independent of tissue type, species, and platform and can be used to predict differentiation status in scRNA-seq data without any prior information.

### Experimental method

As mentioned above, trajectory inference for single-cell analysis provides an avenue to explore the temporal information about cells. The fundamental limitation is that it is only a computational inference and cannot represent the actual existence of dynamic RNA processes. Emerging experimental technologies allow us to distinguish time-resolved phenomena in reality by chemical or biological methods (Table 1).

**Table 1:** Time-resolved single-cell technologies based on experimental method

Experimental Method	Published date	Principle	Advantages and disadvantages	Cultivation method	Research object	Number of cells	Reference
scSLAM-seq	2019 Jul	4sU	Advantages: captures transient and dynamic nature of transcription; applicable for studying single-cell response to perturbations Disadvantages: single cell sample size is too small; extracting mRNA from cells to perform chemical conversion	In vitro	lytic mouse cytomegalovirus infection	110	[32]
NASC-seq	2019 Jul	4sU	Advantages: identifying newly synthesized RNA Disadvantages: single cell sample size is too small; extracting mRNA from cells to perform chemical conversion; restricted to cell populations	In vitro	K562 and Jurkat cells	< 200	[44]
Sci-fate	2020 Aug	4sU	Advantages: using combinatorial indexing to detect more cells; a greater number of genes detected per cell; in situ 4sU (high reaction efficiency and low mRNA loss) Disadvantages: requires complex experimental setups, leading to cell loss	In vitro	Cell cycle and glucocorticoid receptor activation	> 6,000	[45]
scNT-seq	2020 Oct	4sU	Advantages: droplet microfluidics, high-throughput and UMI-based Disadvantages: limited sequencing coverage for scRNA-seq; cannot wash away cell-free RNAs in the droplets	In vitro	Neuronal activation	∼ 55,000	[33]
Well-TEMP-seq	2023 Mar	4sU	Advantages: low cell loss rate, high-throughput and cost-effective; Disadvantages: only for in vitro experiment	In vitro	Anticancer drugs treat colorectal cancer cells	16,000	[34]
scEU-seq	2020 Mar	EU	Advantages: high UMI; high-throughput Disadvantages: requires specialized reagents and procedures; physical separation of new and old transcripts to conduct two libraries	In vitro	Intestinal organoids	5,422	[36]
TrackerSci	2023 Seq	EdU	Advantages: identifying newborn brain cells; revealing cell dynamics Disadvantages: a lesser detection of the quiescent cell population	In vitro/In vivo	brain newborn cells	∼ 800,000	[37]
Fluorescent timer	2019 Feb	dTomato (red); destabilized mNeonGreen (green)	Advantages: offers real-time tracking of gene expression changes during the biological process; from instantaneous changes to long-term process	In vitro/in vivo	Mice and organoids	/	[38]
Zmen-seq	2024 Jan	fluorophore pulse labels	Advantages: exploring biological processes in vivo for multiple days; Disadvantages: the number of fluorescent timers is limited	In vivo	Immune cells in tumor environment (TME)	8,976	[39]
CARLIN	2020 Jun	CRISPR-Cas9	Advantages: Combines CRISPR-Cas9 with scRNA-seq for lineage tracing	In vivo	Genetic lineage tracing	/	[41]

### Metabolic RNA labeling

Metabolic RNA labeling effectively integrates with high-throughput scRNA-seq, addressing limitations pertaining to RNA transcription dynamics [8]. The technology was initially applied at the bulk RNA level [30]. Currently, the most typical metabolic RNA labeling in scRNA-seq is a nucleoside analog called 4-thiouridine (4sU) [31, 32]. The brief technical principle is as follows: After tissue dissociation, the culture medium is supplementary with 4sU. In the process of nascent RNA synthesis, 4sU replaces uracil (U). Reverse transcriptase misread 4sU as cytosine (C). This misreading leads to incorrect pairing, where adenine (A), which normally pairs with U, is replaced by guanine (G). This ultimately results in T-to-C substitutions in the labeled new RNA.

Single-cell, thiol-(SH)-linked alkylation of RNA for metabolic labelling sequencing (scSLAM-seq) pioneered the application of the metabolic labeling method at the single-cell level [32]. This breakthrough identified DEGs in mouse fibroblasts infected and uninfected with cytomegalovirus (CMV) by distinguishing between total, old, and new RNA matrices. The study highlighted a critical insight: most DEGs are primarily detectable in new RNA, eluding detection in analyses of old or total RNA. Additionally, it revealed that interferon and NF-κB varied significantly with different levels of infection, which was a core feature of transcription dynamics at the single-cell level. To address the constraint prohibiting large-scale scSLAM-seq, researchers developed new approaches that integrate high-throughput unique molecular identifier (UMI)-based scRNA-seq analysis with metabolic labeling. It has successfully enabled the acquisition of new/old transcriptomes from thousands of single cells. It has been applied in transcription factor activity and cell state trajectories during neuronal activation [33], as well as the transcriptional dynamics in colorectal cancer cells treated with DNA-demethylating drugs [34]. We anticipate the potential of dynamic transcriptomics application in response to external stimuli (including viral infections and pharmacological interventions), embryonic development, cell differentiation, tumor progression, and immune cell transformation is immense. So far, the application of these technologies in vivo remains unexplored, despite their established utility at the bulk level [35]. It should be emphasized that the combination of scRNA-seq with metabolic labeling in vivo is a feasible avenue that warrants further identification.

In addition to 4sU, Battich et al. presented an approach that utilizes 5-ethynyl-uridine (EU) and click chemistry to separate new and old RNA, thus providing a dynamic view of RNA synthesis and turnover [36]. 5-Ethynyl-2-deoxyuridine (EdU), a thymidine analog, was applied for scRNA-seq and scATAC-seq dynamics [37]. Metabolic labeling-based RNA velocity can accurately recapitulate the dynamics of gene expression.

### Fluorescent timer

The research by Gehart et al. showcased that using fluorescent reporters offers a promising way to reveal real-time-resolved scRNA-seq [38]. *Neurog3* is transiently expressed in enteroendocrine (EE) progenitor cells. They engineered the integration of three independent proteins: NEUROG3, dTomato (red), and destabilized mNeonGreen (green) to study the dynamics of EE cell development. The differing decay rates of mNeonGreen and dTomato enable the measurement of the actual time elapsed since Neurog3 expression in individual cells, determined by the red: green fluorescence ratio. Integrating this approach with scRNA-seq, they have successfully created a real-time-resolved map, revealing the intricate process of EE cell differentiation and development. Recently, Kirschenbaum et al. developed fluorescent-based dynamics scRNA-seq called Zmen-seq in vivo, which uncovered immune dysfunctional trajectories of glioblastoma [39].

### CRISPR/Cas9

Lineage tracing can be deduced by the patterns of mutations shared between cells. The emergence of CRISPR/Cas9 technology facilitates the deliberate introduction of mutations using guide RNAs (gRNAs) [40]. These induced mutations can be applied to readout of lineage histories at the single-cell level [41]. This advanced approach is used to trace cell lineage dynamics during embryonic development and reconstruction [42], as well as to track the growth and dissemination of lung tumor xenografts in mice [43].

### Spatial information

Despite significant advancements in scRNA-seq, current scRNA-seq technologies require isolating cells from the tissues, resulting in the loss of spatial information, which is crucial for investigating intercellular interactions and functional relevance [46, 47]. In recent years, thriving spatial omics technologies have provided robust tools for analyzing spatial information of cells, with spatially resolved transcriptomics acknowledged as Method of the Year in 2020 [48]. Here, we categorize spatial omics technologies into three classes (Table 2) and present the primary technologies and principles (Fig. 3B).

**Table 2:** Primary spatial transcriptomics and spatial multi-omics technologies

Technology	Resolution	Single-cell?	Data type detected	Sample type	Genes/transcripts detected	Reference
ISH-based						
smFISH	Subcellular	Yes	mRNA	Cell	2 genes	[50]
seqFISH	Subcellular	Yes	mRNA	Cell	12 genes	[51]
seqFISH+	Subcellular	Yes	mRNA	Cell	10,000 genes	[53]
MERFISH	Subcellular	Yes	mRNA	Cell, tissue section	140genes, 1,001 genes	[54]
ISS-based						
ISS	Subcellular	Yes	mRNA	Cell, tissue section	31 transcripts	[55]
FISSEQ	Subcellular	Yes	mRNA	Cell, tissue section	8,102 genes	[56]
STARmap	Subcellular	Yes	mRNA	Tissue section	160 ∼ 1,020 genes	[57]
STARmap PLUS	Subcellular	Yes	mRNA, protein	Tissue section	> 20,000 genes	[58]
ROI selection-based						
Geo-seq	Single-cell	Yes	mRNA	FF	> 80,00 genes	[66]
DSP	10 μm	No	mRNA, protein	FF, FFPE	96 genes, 1,412 genes	[68]
Spatial barcode-based						
ST	100 μm	No	mRNA	FF	Whole transcriptome	[72]
10× Visium	55 μm	No	mRNA	FF, FFPE	Whole transcriptome	[74]
Slide-seq	10 μm	No	mRNA	FF	Whole transcriptome	[79]
Slide-seqV2	10 μm	No	mRNA	FF	Whole transcriptome	[80]
HDST	2 μm	No	mRNA	FF	Whole transcriptome	[81]
Stereo-seq	0.22 μm	Yes	mRNA	FF	Whole transcriptome	[82]
Decoder-seq	10 ∼ 50 μm	No	mRNA	FF	Whole transcriptome	[83]
DBiT-seq	10 ∼ 50 μm	No	mRNA, protein	FF, FFPE	Whole transcriptome	[85]
spatial CITE-seq	10 ∼ 50 μm	No	mRNA, epitope	FF	Whole transcriptome	[87]
spatial-ATAC-RNA-seq	10 ∼ 50 μm	No	Chromatin accessibility, mRNA	FF	Whole transcriptome	[86]
spatial-CUT&Tag-RNA-seq	10 ∼ 50 μm	No	Histone modifications, mRNA	FF	Whole transcriptome	[86]
Slide-tags	< 10 μm	No	mRNA, Chromatin accessibility, TCR	FF	Whole transcriptome	[88]

FF, fresh frozen; FFPE, formalin-fixed paraffin-embedded

### Image-based in situ technologies

The image-based in situ technologies evolve from in situ hybridization (ISH) and in situ sequencing (ISS). ISH employs labeled probes to hybridize with target sequences in cells, thereby visualizing the locations of the sequences [49]. ISH-based technologies use complementary fluorescent probes to hybridize with target sequences and detect them. Single-molecule fluorescence ISH (smFISH) is a high-resolution technology that utilizes multiple short oligonucleotide probes coupled with fluorescent moieties to selectively detect transcripts [50], but it is limited by the throughput of detection. The emerging multiplexed FISH technologies address the limitation. Sequential FISH (seqFISH) barcodes the transcripts fixed within cells through multiple rounds of hybridization, imaging, and probe removal, allowing for the detection of the entire transcriptome through four dyes and eight rounds of hybridization [51]. Nevertheless, it suffers optical crowding when profiling an excessive number of transcripts [52]. seq-FISH + is an improved seqFISH, profiling 10,000 genes within individual cells by expanding the barcode base palette to 60 pseudo colors [53]. In addition, it only labels a fraction of transcripts during each hybridization cycle to avoid optical crowding. In another strategy, multiplexed error-robust FISH (MERFISH) can simultaneously image 100 to 1,000 kinds of RNA within individual cells by assigning combinatorial FISH labeling to RNA, followed by successive hybridization and imaging [54].

ISS, first reported in 2013, utilized padlock probes to bind with cDNA produced by reverse transcription, followed by rolling-circle amplification (RCA) to produce rolling-circle products (RCP), which were in situ sequenced and imaged within tissues or cells [55]. Currently, fluorescent in situ sequencing (FISSEQ) and spatially-resolved transcript amplicon readout mapping (STARmap) are two representatives of ISS-based technologies. FISSEQ is an untargeted strategy that uses hexamer primers to reverse transcribe RNA in fixed cells, followed by cDNA circularization, and generates a sequencing library, further improving the detection throughput of ISS [56]. STARmap hybridizes directly to mRNA with a SNAIL probe and then undergoes RCA to obtain DNA amplicons, thereby avoiding reverse transcription [57]. The distinctive feature is its capability to achieve in situ sequencing in three-dimensional (3D) intact tissue. STARmap PLUS further improves detection throughput and is compatible with both transcriptome sequencing and protein detection within the same tissue section, providing a more comprehensive insight into the biological systems [58].

The image-based in situ technologies can achieve subcellular resolution [59]. When combined with single-cell omics technologies, they hold great potential for diverse applications, particularly in neuroscience. For instance, Shi et al. employed STARmap PLUS on 20 central nervous system (CNS) tissue sections and integrated the resulting data with the published scRNA-seq atlas, achieving molecular cell typing of the CNS [60]. Yao et al. integrated MERFISH and scRNA-seq to construct a transcriptomic and spatial atlas of the mouse whole brain and built a platform to visualize these data, providing precious resources for deciphering the complexity of the mammalian brain [61]. Additionally, the combinations can be applied in hematology [62], stem cell research [63], and developmental biology [64].

### ROI selection-based technologies

These technologies employ specific methodologies to precisely select regions of interest (ROIs) from tissue sections for subsequent analysis. Laser capture microdissection (LCM) is a typical technology capable of capturing ROIs at resolutions ranging from cell-population to single-cell via laser cutting [65]. Geographical position sequencing (Geo-seq), an integration of LCM and scRNA-seq, enables the construction of 3D transcriptome atlases, thereby revealing cellular heterogeneity and spatial disparities [66]. Additionally, the integration of high-content imaging, LCM, and multiplexed mass spectrometry can extend single-cell proteomics to intact tissue, significantly improving biological insight [67]. These studies suggest that LCM is a promising technology for isolating ROIs within tissues for multi-omics analysis.

GeoMx digital spatial profiler (DSP), a commercially available technology, is capable of spatially profiling RNA or proteins within the ROIs [68]. It uses a photocleavable (PC) linker to connect oligo sequence (DSP barcodes) with RNA probe or antibody in fixed tissue, selecting ROIs, and releasing barcodes with UV for sequencing. This technology has been used to dissect the heterogeneity of glomerular transcriptional profiler missed by LCM in collapsing glomerulopathy [69], as well as to identify biomarkers associated with immune checkpoint inhibitor (ICI) resistance in non-small cell lung cancer (NSCLC) [70]. However, these technologies are limited by throughput, as each ROI requires individual collection and processing.

### Spatial barcode-based technologies

The core of spatial barcode-based technologies is capturing transcripts within tissues or cells using spatial barcodes (DNA oligos) array on glass slides [71]. Subsequently, library preparation and next-generation sequencing (NGS) are performed.

Spatial transcriptomics (ST) was a milestone achievement, carrying epochal significance for spatially resolved transcriptomics [72]. It innovatively captured polyadenylated RNA on spot-equipped slides, with each spot containing unique spatial barcodes, ensuring that each transcript was precisely mapped back to its respective spot through the spatial barcode [73]. ST was first applied in adult mouse olfactory bulb and human breast cancer, achieving RNA sequencing while preserving two-dimensional spatial information. 10× Genomic improved it and released 10× Visium, which possessed a higher spatial resolution [74]. It has been commercialized and extensively employed across diverse fields such as developmental biology [75], cancer biology [76], as well as neuroscience [77, 78]. For instance, Olaniru et al. applied the integration of scRNA-seq with 10× Visium to the developing human fetal pancreases, analyzing the differentiation and maturation processes of various cell types [75]. Galeano Niño et al. applied 10× Visium to oral squamous cell carcinoma and colorectal cancer, identifying the identity and in situ position of the microbial communities within the tumors [76]. Hasel et al. integrated bulk RNA-seq, and scRNA-seq with 10× Visium, uncovering spatiotemporal heterogeneity in the response of different astrocyte subsets to inflammation in the brain [78]. These applications suggest that 10× Visium is a highly promising technology for dissecting spatial information.

Slide-seq captures transcripts on a slide equipped with random 10-µm DNA-barcoded beads and gains the positions of barcodes via in situ indexing, enabling spatial transcriptomic analysis at a near-cellular resolution [79]. Stickels et al. optimized Slide-seq and reported the highly sensitive Slide-seqV2, leading to a tenfold increase in RNA capture efficiency [80]. High-definition spatial transcriptomics (HDST) utilizes a split-pool approach to generate a dense and barcoded bead array, capturing RNA at a 2-µm resolution [81]. Recently developed spatial enhanced resolution omics-sequencing (Stereo-seq) utilized a randomly barcoded DNA nanoball patterned array chip to achieve single-cell resolution and has been employed to delineate the spatiotemporal transcriptomic landscape of mouse organ development [82].

However, the aforementioned spatial barcode-based technologies suffer several constraints, such as multicellular resolution, low sensitivity, and the necessity for sequencing to obtain positional indexing. To address these constraints, Cao et al. presented a dendrimeric DNA coordinate barcoding design for spatial RNA sequencing (Decoder-seq) [83]. The central innovation was to employ a microfluidics-assisted combinational barcoding approach to create high-density spatial barcode arrays on a 3D dendrimeric nanosubstrate, enabling cost-effective, highly sensitive, near-cellular resolution spatial transcriptomics research. They utilized it to spatially resolve the distribution of low-expressed olfactory receptor genes and accurately depict the spatial single-cell landscape of the hippocampus.

Recently, spatially resolved multi-omics has been recognized as one of the noteworthy technologies in 2023 [84]. Fan’s group pioneered spatial multi-omics technology. They innovatively employed two orthogonal chips equipped with parallel microfluidic channels to deliver DNA barcodes to tissue sections, developing the first spatial multi-omics technology, deterministic barcoding in tissue for spatial omics sequencing (DBiT-seq), enabling simultaneous detection of the whole transcriptome and 22 proteins [85]. Furthermore, the group extended this approach to spatial ATAC-RNA-seq and spatial assay of cleavage under targets and tagmentation and RNA sequencing (spatial CUT&Tag-RNA-seq) [86], which achieve co-profile of epigenome and transcriptome, as well as spatial co-indexing of transcriptomes and epitopes for multi-omics mapping by highly parallel sequencing (spatial CITE-seq) [87], which is capable of co-mapping transcriptome and epitope. A very recently developed technology, Slide-tags, can label single nuclei with spatial barcodes and isolate them for multi-omics analysis [88]. Altogether, these spatial multi-omics technologies provide more comprehensive biological information, deepening our understanding of the intricate spatial and molecular interactions in the field of life science and biomedical research.

### Genome

Although genomes are generally considered to be stable, there is still a small possibility of genetic mutations occurring with each DNA replication. Single-cell whole-genome sequencing (scWGS) is capable of elucidating genomic heterogeneity and can therefore be utilized to analyze genomic mutations in single cells. This technology involves the isolation of individual cells or nuclei followed by whole-genome amplification (WGA), library preparation, and sequencing [89]. It has been applied to uncover somatic mutations in multiple cell types, such as human bronchial epithelial cells [90], and B lymphocytes [91], offering novel insights into the pathogenesis of diseases.

Single nucleotide polymorphism (SNP), a common form of genetic variation, is caused by the transition, transversion, insertion, or deletion of individual bases. Expression quantitative trait loci (eQTL) analysis can profile genetic variations (especially SNP) that affect gene expression, offering enhanced insights into the relationship between genetic variants and gene regulation [92, 93]. The integration of scRNA-seq with eQTL was first reported in 2018 [94]. Kang et al. utilized multiplexed droplet scRNA-seq to profile eight immune cell populations from 23 donors and subsequently conducted eQTL analysis, identifying 32 cis-eQTLs, 22 of which were cell-specific. Recently, Ding et al. constructed the first integrated human sceQTL database, which comprises ∼ 16 million SNPs and ∼ 0.69 million sceQTLs [95], providing a valuable resource for disease susceptibility gene discovery.

In addition, multiple multi-omics technologies for integrative analysis of genome and transcriptome have been developed, among which genome and transcriptome sequencing (G&T-seq) [96] and gDNA-mRNA sequencing (DR-seq) [97] stand out as two representative technologies. G&T-seq utilizes oligo-dT-coated beads to isolate mRNA and DNA, which are subsequently amplified and subjected to whole transcriptome sequencing and WGS, respectively. Whereas DR-seq employs a preamplification-and-separation strategy to decouple DNA and mRNA molecular analytes, thereby avoiding physical nucleic acid separation before amplification. Therefore, compared with G&T-seq, DR-seq has a lower cross-contamination rate and a higher recovery rate. Collectively, these technologies hold vast potential for deciphering genetic variations and their impacts on gene expression.

### Epigenome

Epigenomics aims to explore how chemical modifications and spatial structure alterations of the genome affect gene function and expression regulation. Deciphering the epigenomic features such as DNA methylation, chromatin accessibility and histone modifications at the single-cell level allows us to study cell lineages and differentiation states [98].

In eukaryotes, the most common DNA methylation occurs on the fifth carbon atom of C within the CpG island, yielding 5-methylcytosine (5-mC). Bisulfite conversion is the gold standard for DNA methylation analysis [99]. The primary principle is that methylated C in DNA remains unchanged after treatment with bisulfite, whereas unmethylated C is converted to U [100]. After PCR amplification and high-throughput sequencing, the bases that methylated can be ascertained by comparison with reference sequences. Single-cell DNA methylation sequencing based on bisulfite conversion and NGS can be categorized into single-cell reduced representation bisulfite sequencing (scRRBS) [101] and single-cell whole-genome bisulfite sequencing (scWGBS) [102], which achieve single-base resolution. These technologies play crucial roles in investigating cell differentiation and development. The first single-cell multi-omics technology achieving co-profile of the DNA methylome and transcriptome is single-cell methylome and transcriptome sequencing (scM&T-seq) [103], which utilizes G&T-seq to separate and amplify genomic DNA and mRNA from the same single cell and applies scBS-seq [104] to generate DNA methylation data. In addition, single-cell triple omics sequencing (scTrio-seq), which co-profiles the genome, DNA methylome, and transcriptome through scRRBS and WGS, has been applied to 25 cancer cells and identified two distinct subsets [105]. However, one constraint of these technologies is that bisulfite conversion involves intense chemical reactions that can lead to significant DNA degradation and consequent loss of information.

Chromatin accessibility, a key epigenetic feature, plays a pivotal role in regulating gene expression by allowing transcriptional machinery to interact with regulatory elements, thereby facilitating the initiation or suppression of gene transcription in open chromatin regions [106]. Recently a variety of technologies have been developed to interrogate chromatin accessibility [107–109], whereas ATAC-seq barges to the forefront as a landmark breakthrough that utilizes the Tn5 transposase to fragment open chromatin and labels the genome with sequencing adaptors [110]. Subsequently, the labeled genome undergoes PCR amplification and sequencing (Fig. 2F). Two methods of scATAC-seq were developed in 2015, which enabled the exploration of chromatin accessibility at the single-cell level. The first one utilized a programmable microfluidics platform to capture single cells, followed by Tn5 transposase tagmentation and library amplification with cell-identifying barcoded primes [111]. The other one utilized an integrative method combining combinatorial cell indexing with ATAC-seq to analyze chromatin accessibility within over 15,000 cells [112]. 10× Genomics developed a Chromium platform and applied it to scATAC-seq, which combined Tn5 transposase tagmentation within bulk nuclei and single-nuclei isolation through the droplet system [113]. Currently, the integration of scRNA-seq and scATAC-seq has been applied to explore the regulation of human developmental hematopoiesis [114], as well as to find potential therapeutic targets for clear cell renal cell carcinoma [115].

Histone modifications are chemical modifications that occur at specific sites on histone molecules, thereby affecting chromatin structure stability and gene expression regulation [116]. Chromatin immunoprecipitation sequencing (ChIP-seq) is a common method to profile histone modifications [117]. Single-cell ChIP-seq (scChIP-seq) tags nucleosomes with barcodes via a droplet microfluidic platform before conventional ChIP-seq, and it was employed to interrogate the chromatin landscapes of breast cancer [118]. However, ChIP-seq has a high demand for experimental samples. To address the obstacle, Kaya-Okur et al. introduced CUT&Tag, which utilizes Protein A-Tn5 transposase to cleave the DNA sequences bound by targeted protein and integrates sequencing adapters with the cleaved sequences [119]. On this basis, single-cell CUT&Tag (scCUT&Tag) integrated CUT&Tag with 10× Genomics scATAC-seq protocol, enabling the investigation of histone modifications at single-cell resolution [120]. It was applied to explore the histone modification features of regulatory elements and gene bodies in the central nervous system cells of mice. Recently, some multi-omics technologies have been developed, such as Paired-Tag [121] and combined assay of transcriptome and enriched chromatin binding (CoTECH) [122], which utilize a combinatorial barcoding strategy, achieving co-profile of histone modifications and transcriptome in single cells.

### Cellular protein and epitope

Single-cell proteomics is a more nascent field. Transcriptomic features may not exhibit a comprehensive snapshot of cellular heterogeneity since similar gene expression profiles may be identifiable in other modalities that are simultaneously measured. Indeed, the transcriptomes and proteomes represent distinct molecular modalities, such as post-translational modifications that cannot be captured by transcriptomics. It’s crucial to simultaneously identify the transcriptome and protein abundance at the single-cell level. Mass spectrometry-based single-cell proteomics (scMS), achieving a detection depth of about 1,500 ∼ 2,500 proteins, is the most successful and extensively discussed in this review [123]. Furthermore, imaging-based approaches address the issue of spatial distribution [123]. Integrated with the burgeoning scRNA-seq technology, spatial resolution scMS was applied to explore pivotal modalities of the skin dermal fibroblast cells [124]. The challenge of quantifying proteins in sequencing is addressed by leveraging the binding of specific antibodies linked to oligonucleotide for translation and amplification, thus overcoming the limitations of existing RNA-seq methods, which are unable to directly measure proteins. Cellular indexing of transcriptomes and epitopes by sequencing (CITE-seq), one of the most widely used methods that sequence cellular surface protein abundance via oligonucleotide-conjugated antibodies, combining protein-level and RNA-level insights within single-cell analyses (Fig. 2C) [125, 126]. Furthermore, they developed the applicable strategy of integrated multi-omics single-cell data to adjust for the varying multi-omics quantifications. They constructed a comprehensive atlas on the circulating human immune system based on the multimodal definition [7]. Trzupek et al. discovered a novel subset of T regulatory cells (Tregs) with significantly upregulated CD80 and CD86, as revealed by antibodies-based sequencing. Moreover, their data indicated a low correlation between RNA and protein levels [127].

Proteins and other epitopes in cells are constantly dynamic changes in spatiotemporal processes, thus adding information about proteins or other epitopes to scRNA-seq can accelerate the understanding of cellular states and functions. Recently, Glycan epitopes on the surface have been described as the specific cell states and types, some studies have also focused on it [128–130]. SUrface-protein Glycan And RNA-seq (SUGAR-seq) revealed unique surface glycan profiles in tumor-infiltrating lymphocytes (TILs) [128]. Yu et al. have revealed that N-acetyllactosamine (LacNAc), a glycans related to immune receptor signaling, serves as a distinct indicator for discerning the glycolytic activity and effector function of CD8^+^ T cells [129].

A series of research has proved that cellular epitope information can reveal the phenotypes that could not be detected by scRNA-seq alone. Although existing technologies empowered scRNA-seq analysis by providing rich surface protein and epitope resources, it seems difficult to establish high-throughput single-cell proteomics in parallel [131].

### Immune Repertoire

Immune repertoire (IR) encompasses the diversity of T/B cells in a particular environment, indicative of the capacity of the immune system to respond to external stimuli at a particular moment. T/B cells, as the primary cell populations in the specific immune system, are at central focus in immunological research, where deciphering their characteristics and functions remains key to delineating the immune microenvironment. scTCR-seq and scBCR-seq enable the determination of the full-length or complementarity-determining region 3 (CDR3) gene sequences related to TCR and BCR, respectively, at the single-cell level. It complements the limitation that scRNA-seq only obtains the transcriptome landscape, providing deep insights into the behavior of T/B cell populations, and enabling researchers to understand and possibly manipulate these responses for therapeutic purposes. The main directions of application are TME research, the evaluation and monitoring of immune/infectious diseases, and TCR, BCR, and antibody screening.

### TCR

It is well-known that T cells recognize the peptide-loaded major histocompatibility complex (pMHC) presented by antigen-presenting cells via the TCRs, thereby triggering the subsequent immune response to kill cancerous or infectious cells [132]. Deciphering TCR repertoire in varying situations is the foundation for understanding mechanisms, diagnostics, and developing new vaccines.

TCR is composed of α chain (TRA) and β chain (TRB), which are produced by combinatorial rearrangement of gene segments: variable (V), diversity (D) (exclusive to the β chain), joining (J), and constant (C). This recombination process results in a vast diversity of TCR repertoire, predominantly determined by the hypervariable CDR3 [133]. Given that the probability of identical rearrangements occurring in the absence of selection pressure is extremely low, TCR sequencing has been identified as a valuable indicator for antigen-driven clonal expansion, reflecting antigen specificity and response.

The history of high-throughput TCR sequencing has been introduced thoroughly in this review [134]. The most widespread application involves simultaneous scRNA-seq and scTCR-seq. In this method, cDNA is partly used for the enrichment of TCR, while the other is utilized to construct gene profiles (Fig. 2A). This technology has been extensively commercially adopted by multiple companies. In RNA, TRA, and TRB sequencing, sequences shared with the same barcode are computationally inferred as a pair. However, a current limitation of this technology is the possibility of missing a chain and incorrectly matching with more than two TCR sequences.

A series of heavyweight studies on the TILs have exhibited the T cell and TCR clonotype diversity, constructing the landscape of T cell heterogeneity and dynamics in the TME [135–137].

In routine analysis, the primary objective is to map and analyze the diversity of clonotypes or CDR3. Additionally, clonotype overlap represents another critical dimension, empowering the characterization of TCR sequences shared across different tissues or tracking of key clonotypes during therapeutic interventions. From the excellent work of Zhang’s group, they pioneered the development of multiple indicators such as single T cell analysis by RNA sequencing and TCR tracking (STARTRAC) (for clonal expansion, tissue migration, and state transition), R_o/e_, and OR (for tissue distribution) to assess the status of TCR [135–137].

It is crucial to explore the differentiation of TCRs in cell types/ tissues/ conditions. Chen et al. developed TCRdb, a comprehensive TCR database, that aids in identifying the states and functions of specific sequences [138]. Another significant challenge is unraveling the binding between TCR-pMHC in the context of infectious diseases or tumors. Advanced bioinformatic algorithms accelerate research breakthroughs in this field. Among these tools, NetMHCpan stands out in predicting the binding affinity between peptides and MHC-I/II, respectively [139]. Additionally, Gao et al. have developed a computational tool to predict TCR-peptide binding via a machine learning method [140].

In all, scTCR-seq and associated analyses enhance the exploration and application of TCR sequencing. They facilitate the exploration of mechanisms, helping cancer early-stage diagnosis, treatment selection, and prognosis prediction, and designing engineering therapeutic strategies.

### BCR

Similar to TCR, BCR consists of immunoglobulin heavy chains and light chains​​, the diversity of it is produced by gene rearrangement of V(D)J gene. Based on its structure, BCR plays a central role in the adaptive immune response by recognizing specific antigens, which trigger B cell activation, subsequently producing antibodies [141]. Around a decade ago, paired scBCR-seq was introduced [142, 143]. scBCR-seq allows for a thorough analysis of BCR repertoires, providing crucial information about their diversity and function at the single-cell level. More recently, Ian et al. challenged limited information about BCR-seq to antigen specificity and developed LInking B-cell Receptor to Antigen specificity through sequencing (LIBRA-seq) to confirm for HIV-and influenza-specific antibodies [6]. Combined with scRNA-seq, scBCR-seq was applied for producing cell atlas [144], identifying different neutralizing antibodies [145], conducting vaccine studies [146], etc., and proved invaluable in studies investigating the dynamics of the immune system. From a clinical perspective, it helps diagnose and monitor tumor immunology [144], infectious diseases [145, 147], autoimmune diseases, and immunodeficiencies.

### Microbiome

Microbiome refers to the collection of microorganisms living in a particular environment. The main technologies in the microbiome field are metagenomic sequencing and 16 S rRNA sequencing [148–150], updating our understanding of microbial community on a more microscopic level (Fig. 2D). Recently an advanced technology called barcoding bacteria for identification and quantification (BarBIQ), achieved precise single-base in 16 S rRNA sequencing through the use of unique barcodes and a droplet-based approach [151]. Microbiomes can be assessed at the single-cell level, addressing the issue where traditional methods may obscure the simultaneous measurement of cell counts for each type of bacteria. In the context of scRNA-seq, although it has become a transformative technology for profiling gene expression levels in thousands of eukaryotic cells, challenges such as the low volume of RNA, no polyadenylate tail in bacterial RNA, and resistant cell wall have long hindered the adaptation of scRNA-seq technology to microbes. To overcome these obstacles, Kuchina et al. introduced microbial split-pool ligation transcriptomics (microSPLiT), a technology that can identify the scarce subpopulations of cells down to a minuscule proportion of 0.142%, which was crucial for revealing rare cellular states that are significant from a physiological perspective [152]. Similarly, BacDrop was developed for bacterial scRNA-seq, identifying bacterial types and quantifying the number of specific types of cells [153]. Besides, Microbe-seq applied microfluidic-droplet operation and bioinformatic analysis to obtain the genomes of numerous microbes with single-cell resolution, and most single-amplified genomes had a purity of over 95% [154].

The intratumor microbiome has emerged as a novel and rapidly evolving research frontier, with the discovery of microorganisms in various cancer types, including in some organs traditionally considered to be sterile, primarily in gastrointestinal cancers [155–157]. However, the subtle relationship between them and cancer remains unclear, sequencing technologies may be an effective solution. Galeano et al. introduced invasion-adhesion-directed expression sequencing (INVADEseq) targeting a conserved area of the bacterial 16 S rRNA, enabling the effective creation of cDNA libraries containing bacterial transcripts derived from human cells associated with bacteria. The technology is crucial to uncovering the complexity of microbiota interactions within tumor tissues [158, 159].

Another interesting bioinformatical approach called Single-cell Analysis of Host-Microbiome Interactions (SAHMI) [160], is quite fascinating as it systematically extracts real microbial signals and quantifies microbiome profiles directly from mammalian host sequencing data. SAHMI advances a similar method into the realm of single-cell analysis, enabling the identification of microbial species related to specific cell types and uncovering the relationship between microbial and transcriptome profiles, facilitating a deeper investigation into their contribution to intercellular communication networks [160].

### Metabolome

Metabolome focuses on the biochemical reactions within cells, encompassing the collection of all metabolites of tissue in a specific physiological period and metabolic features (Fig. 2E). The field of single-cell metabolomics marks a pivotal era in unraveling the complexities of cellular processes at the individual cell level. Mass spectrometry (MS) [161–164] and nuclear magnetic resonance (NMR) spectroscopy [165] are widely utilized technologies for metabolomics analysis. Recent advancements have enabled the analysis of single-cell metabolomics and even at the level of single-organelle [166]. A comprehensive overview of the history and advances of metabolomic methodologies is provided in this review [167], but this review refrains from further elaboration. Importantly, it remains a challenge to combine metabolomics with other single-cell multi-omics technologies given the limited sample size of single cells and the destructive quality of analyses and sequencing. In this respect, advanced technologies that possess the potential to integrate with other omics-based technologies are warranted.

### Cell-cell interactions (CCIs)

With the advancement of bioinformatic analysis, tools developed based on scRNA-seq for inferring cell-cell communications, such as CellChat [168], CellPhoneDB [13], NicheNet [169], and CellCall [170], have become mainstreams. However, these methods of inferring CCIs through algorithms are influenced by parameters and do not necessarily represent actual occurrences. The application of chemical biology tools in medical research is expanding, with proximity labeling technologies that may become game changers for reflecting real-world CCIs [171–174]. A groundbreaking research reported that FucoID, a chemical biology tool for capturing tumor antigen-specific T cells through dendritic cell interactions using fucosyltransferase (FT) [171]. Based on this, they further developed an advanced platform for T cell-cancer cell and B cell-dendritic cell (DC) interactions adapted for complex systems [173]. This promising technology has been effectively integrated with RNA-seq and flow cytometry. Its combination with sing-cell technology is bound to reflect CCIs from a real perspective and could offer real insight into the exploration of tumor-reactive TCR.

### Perspective in application in biological research

**Fig. 4:**
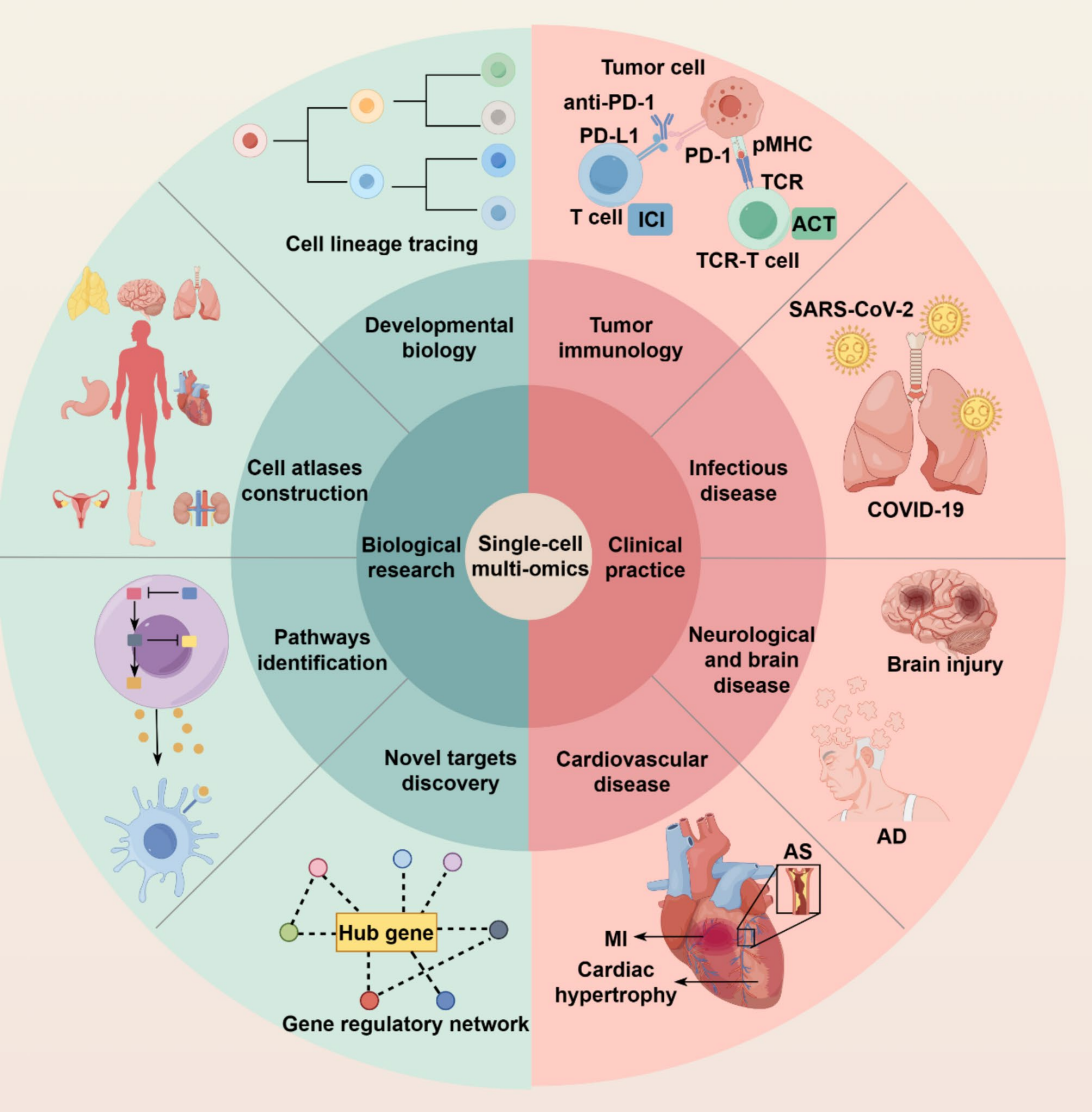
Applications of single-cell multi-omics in biological research and clinical practice

Currently, single-cell multi-omics technologies are rapidly evolving, offering robust tools for depicting intricate cellular landscapes. They have been applied to a diversity of fields including, but not limited to, cell atlas construction, developmental biology, pathways identification, and novel targets discovery, with remarkable achievements (Fig. 4).

### Cell atlases construction

Constructing cell atlases is a common function of single-cell multi-omics technologies. Cell atlases, as one of the seven noteworthy technologies in 2024 [175], can display detailed information about various cell types within different organisms, allowing for the characterization of cellular diversity [176], the analysis of cellular heterogeneity [177], as well as the discovery of novel cell types [178]. In recent years, the great advances in single-cell multi-omics technologies have enabled scientists to characterize various molecular information within individual cells, deepening our understanding of different cell types.

The largest cell-atlas initiative, the Human Cell Atlas was launched in 2017, aiming to integrate single-cell omic data into comprehensive atlases, thereby enhancing our understanding of cell development, physiology, and CCIs [74]. Currently, with the joint efforts of scientists, cell atlases of a wide range of organs and disease tissues have been constructed [179, 180]. By comparing the cell atlases of healthy and diseased states, we can uncover the mechanisms underlying diseases and advance their diagnosis and treatment [181]. In addition, single-cell multi-omics technologies can be applied to construct cell atlases of organoids for fundamental research as demonstrated in research that constructed a human brain organoid development atlas by co-profiling of transcriptome and chromatin accessibility to investigate the regulation of cell fate decisions [182]. Therefore, constructing comprehensive cell atlases with single-cell multi-omics technologies will have profound impacts on biological research and human health.

### Developmental biology

A principal application of single-cell multi-omics technologies in developmental biology is lineage tracing, which aims to track the progeny of individual cells to investigate cellular differentiation trajectories [183, 184]. Compared with traditional methods to study cell lineage with heritable tags or naturally occurring somatic mutations [185], single-cell multi-omics technologies provide powerful tools for delineating comprehensive lineage relationships and diverse cellular states. A newly developed multi-omics technology, single-cell Regulatory multi-omics with Deep Mitochondrial mutation profiling (ReDeeM), utilizes naturally occurring mitochondrial DNA mutations as barcodes for lineage tracing while analyzing transcriptome and chromatin accessibility [186]. It was employed to construct a phylogenetic tree of human hematopoiesis, revealing the clonal architecture, functional heterogeneity, and age-related changes of hematopoietic stem cells. Another single-cell lineage-tracing technology, Camallia-seq, enables integrative analysis of chromatin accessibility, DNA methylation, transcriptome, as well as cell lineage information, bringing new insights into how cell fate decisions are regulated and how cell identities are maintained under different modalities [187].

Additionally, single-cell multi-omics technologies have been employed for multiple stages of embryonic development, including preimplantation [188, 189], implantation [190], gastrulation [191], and early organogenesis [192], to explore cell fate decisions of embryonic development from multiple dimensions, thereby providing a paradigm to decipher the molecular programs of tissue architecture and cellular organization [193]. To comprehensively understand embryonic development, it is essential to explore the spatial information of cells, as it is one of the key factors determining cellular identity. Integrated analysis of single-cell and spatial transcriptomics can be applied to embryonic development, as in a study that precisely characterized human embryonic limb development over time and space [194].

Altogether, with rapid advances in lineage tracing, single-cell multi-omics, and spatial transcriptomics, we will address fundamental questions of developmental biology, achieving a better understanding of cell differentiation and development.

### Pathways identification

The currently thriving single-cell multi-omics technologies are applied to identifying signaling pathways required for cellular function, shedding light on the mechanisms of multiple key pathological processes [195–199]. Fan et al. performed an integrative analysis of cervical squamous cell carcinoma (CSCC) utilizing scRNA-seq, Stereo-seq, and spatial proteomics, identifying eight meta-programs (MP) [195]. Notably, they revealed that MP6 tumor cells interact with cancer-associated fibroblasts (CAFs) to shape an immune exclusionary microenvironment via the FABP5-mediated transforming growth factor β (TGFβ) pathway. Han et al. applied scRNA-seq and scATAC-seq to characterize neuroendocrine prostate cancer (NEPC) cells, identifying the KIT pathway, which can be activated by FOXA2 to maintain cancer cell proliferation [196]. Inhibition of KIT can be a potential strategy for the treatment of NEPC. In addition, integrated analysis of epigenomics and transcriptomics was applied to investigate the transcriptional dynamics of the fibrotic kidney, revealing that the TF Nfix could regulate the expression of the apoptosis-related gene Ifi27 [198]. The Nfix-Ifi27 pathway was also identified, which can cause kidney fibrosis by promoting apoptosis. Therefore, leveraging single-cell multi-omics technologies to identify signaling pathways offers crucial insights into the mechanisms of diseases, opening up promising avenues for the development of innovative therapeutic strategies.

### Novel targets discovery

The single-cell multi-omics technologies can integrate information at multiple levels to construct a comprehensive gene regulatory network and elucidate the regulatory and causal relationships between various molecules, thus holding great potential in discovering novel targets [200]. Olatoke et al. performed an integrative scRNA-seq/single-nucleus ATAC sequencing (snATAC-seq) analysis on lymphangioleiomyomatosis (LAM) to construct a HOX-PBX gene regulatory network that controlled the survival of LAM cells, thereby providing potential therapeutic targets for LAM [201]. Pozniak et al. integrated single-cell transcriptomics with spatial transcriptomics and proteomics to investigate melanoma, revealing a TCF4-dependent regulatory network, which orchestrated multiple transcriptional programs leading to immunotherapy resistance [202]. Targeting TCF4 can enhance the sensitivity of melanoma to ICI and targeted therapy. In another study, scRNA-seq and ST were applied to characterize the cellular composition and spatial structure of multiple primary lung cancers (MPLCs), finding that TNFRSF18 was highly expressed in T&NK cells within tumor tissues [203]. TNFRSF18 has been demonstrated to be associated with non-response to anti-PD-1 therapy in lung cancer [204]. Thus, it is anticipated that single-cell multi-omics and spatial technologies will create a more comprehensive framework, providing unprecedented opportunities to discover novel targets for disease intervention.

## Perspective in applications in clinical practice

The advent of single-cell multi-omics technologies has added breadth and depth to understanding a battery of complex diseases and their pathology including neurological disease, immune disorders, oncology, and others. Within this section, we delve into the applications of single-cell multi-omics across diverse fields, underscoring its transformative impact on clinical practice.

### Tumor immunology

Cancer therapies are continually being developed and optimized, most of which can remodel the TME. As knowledge of the immune system improves, new immunotherapies, represented by ICIs and adoptive cell therapy (ACT), are emerging. The capability of single-cell multi-omics technologies to uncover the intricate interactions between the diverse cells and cancer cells sheds light on the heterogeneity and complexity of TME, positioning them as promising tools in the exploration of cancer treatment strategies.

### ICIs

How do T cells react during ICI therapy? Paired scRNA-seq and scTCR-seq might provide deep profiling. One study from ICI therapy for NSCLC, deciphered tumor-specific T cell clonotype feature, regional distribution, and temporal persistence during ICI therapy [205]. Another study from Qiu et al. utilized scRNA-seq, paralleled with scTCR/BCR-seq to elucidate the treatment response of Epstein-Barr virus (EBV)-associated gastric cancer. Notably, re-emerged clonotypes in ISG-15^+^CD8^+^ T cells after treatment among EBV (+) patients were detected and associated with effector T population expressing CXCL13 in responsive EBV (+) tumor, indicating their significant importance in tumor immunochemotherapy response [25]. David Y. Oh et al. assessed the transcriptome characteristics of T cells and paired TCR from human bladder tumors. Unexpectedly, the typical CD8^+^ T cell states were unchanged in tumor and normal tissues, while cytotoxic CD4^+^ T cells showed the opposite. They also managed to predict the therapeutic effect of anti-PD-L1 in bladder cancer patients based on CD4 signature score [206]. A study combined scRNA-seq, TCR-seq, and ATAC-seq for integrated analysis, suggesting that TdLN-T_TSM_ cells are primary memory T cells that respond to ICI treatment, representing adoptive these cells a promising immunotherapy approach [207].

### ACT

With the development of engineered T cells, ACT therapies, represented by T cell receptor-T cell (TCR-T), Chimeric antigens receptor-T cell (CAR-T), and TILs have reshaped the landscape of tumor treatment. Tumor-specific TCRs can recognize tumor-specific antigens, which provides a solid foundation for the development of TCR-T therapy. Recent research found CXCL13, CD200, and ENTPD1 as unique markers for tumor antigen-specific T cells using scRNA-seq and scTCR-seq. On this basis, developed tumor antigen-specific TCR-T cell therapies have shown significant therapeutic efficacy in autologous patient-derived xenograft (PDX) tumors [208]. Because of the unclear molecular mechanisms of resistance to CAR-T therapy in acute lymphoblastic leukemia (ALL), Bai et al. integrated scRNA-seq and CITE-seq to compare responders and CD19-positive relapse patients, during which they confirmed lack of T_H_2 functionality might be the cause of relapse in CAR-T treatment [209]. In addition, the researchers conducted a single-cell multi-omics (RNA, TCR, and CITE-seq) study in TILs from NSCLC patients to establish a neoantigen-targeted T-cell signature characterized by the frequency of clonotypes along with the levels of CD39 protein and CXCL13 RNA. Utilizing this signature, they were able to detect neoantigen-reactive TCRs with a success rate [210].

### Host-microbe interactions

A series of thrilling advancements in the interaction between the human body and microorganisms have fully illustrated the protective or pathogenic effects of bacteria, scRNA-seq undoubtedly is the promising method to answer the open questions [211]. Integrated 16 S rRNA-seq and scRNA-seq analysis have been widely utilized in microbiota gastric cancer [212], pancreatic injury [213], cholangiocarcinoma [214], etc., exploring the potential relationship between microbe and host cell types by complementing the composition of microbial communities and host cell and genetic information. A groundbreaking study integrated 16 S rRNA and scRNA-seq to reveal that *Streptococcus anginosus* promotes gastric tumorigenesis [215]. Jia et al. integrated 16 S rRNA-seq with single-cell transcriptomics, TCR-seq, and ATAC-seq to reveal that IPA activates progenitor-exhausted CD8^+^ T cells through H3K27 acetylation modification [216]. It is worth mentioning that Chai et al. employed the Kraken method [217, 218] to process scRNA-seq to obtain the bacterial population corresponding to specific cell types [214].

### Infectious diseases

In the realm of infectious diseases, the technologies shed light on host-pathogen dynamics, immune responses, and advanced therapeutic strategies, especially in COVID-19 [145, 146, 219–223]. Su et al. employed single-cell multi-omics (RNA, CITE, TCR/BCR, etc.) to observe unique dynamics in the behavior of specific CD8^+^ T cells during the recuperation phase from COVID-19, among patients suffering from gastrointestinal sequelae [219]. Besides, the method is of great significance for the development and evaluation of vaccines. Through scRNA/TCR/BCR-seq, Peng et al. systematically profiled the immune landscape after vaccinating lipid nanoparticle-mRNA [146]. A study focused on the breakthrough infection and pan-variant antivirals, and they successfully identified elite neutralizing antibodies (nAbs) repertoire using scRNA/BCR-seq of B cells, which showed strong neutralizing activity targeting numerous variants [145]. In addition, it has been found that the crosstalk of specific T cells and B cells following COVID-19 vaccine treatment [220, 221].

### Cardiovascular disease

An in-depth exploration of cardiac disease using single-cell technologies contributing to predicting disease, therapeutic target discovery, and stratifying patients [224–226]. The work from Kanemaru et al. employed sc-RNA-seq, single-nucleus RNA sequencing (snRNA-seq), snATAC-seq, and spatial transcriptomics, paving the way for the anatomy and immunology of the heart [227]. Delgobo et al. focused on the transgenic T cell receptor (TCR-M) cells and myocardial infarction (MI). Using scRNA/TCR-seq, they elucidated TCR-M cells expressing Treg markers like Foxp3, Il2ra, and Ctla4 and suppressed cardiac immune responses post-MI and improved cardiac function [228]. A hypertrophy study applied multiple-dimensional approaches including epigenetic and morphological analysis to the mechanism of pressure overload [229]. In addition, a study employed CyTOF, scRNA-seq, and CITE-seq to decipher the immune landscapes in the plaques of atherosclerosis (AS) and uncover immune alterations related to clinical cardiovascular events, suggesting potential avenues for AS treatment [230].

### Neurological and brain disease

The high complexity of brain cells requires advanced single-cell multi-omics technologies to resolve the basic gene regulation both in healthy and neuropsychiatric brain tissues [231]. A 2024 review comprehensively compiled studies on Alzheimer’s disease (AD) using transcriptomics, metabolomics, and other advanced technologies, summarizing mechanisms and targets of sex differences in AD progression [232]. Notaras et al. performed an integrative analysis of transcriptome and proteome on schizophrenia organoids to identify two disease-associated factors (BRN2 and PTN). Both BRN2 and PTN promoted neurogenesis, while PTN also inhibited apoptosis. Besides, the depletion of BRN2 and PTN can lead to schizophrenia through different mechanisms [233]. In addition, Ji et al. demonstrated that Glutaminase 1 deficiency in forebrain neurons can lead to autism spectrum disorder-like behaviors by single-cell multi-omics analysis [234].

As mentioned earlier, the combination of single-cell sequencing and spatial transcriptomics holds vast promise for the study of brain diseases. Li et al. employed scRNA-seq and spatial transcriptomics to identify two distinct microglial subclusters (ICAM and IPAM). ICAM was related to ischemia, exhibiting pro-inflammatory characteristics. In contrast, IPAM, associated with the ischemic penumbra, with inflammation-alleviating and neuroprotective features. Thus, they reported that targeting specific microglial subclusters is a promising therapeutic strategy for ischemic stroke [24]. Similarly, Han et al. combined scRNA-seq with spatial transcriptomics to identify LGALS9-CD44 as a crucial pathway after ischemic injury. LGALS9 and CD44 exhibited opposite effects, in which upregulation of LGALS9 favored recovery from post-ischemic injury, whereas knockdown of CD44 diminished the therapeutic effect of LGALS9 [235]. These applications suggest that single-cell multi-omics technologies are eminently prospective for investigating neuropsychiatric disorders as well as brain injuries, providing unprecedented opportunities to decipher the complexity of the brain.

In addition to the applications above (Table 3), we anticipated that single-cell multi-omics and paired bioinformatics tools would provide a fundamental framework for the research on a variety of complex diseases or biological processes, including autoimmune diseases [236], aging [237], spermatogenesis [238], and others [239, 240].

**Table 3:** Applications in biological research and clinical practice

Technology	Diseases	Source material	Main findings	Reference
Cell atlases construction				
scRNA-seq, spatial transcriptomics	/	Human	The study highlighted the transformative potential of single-cell and spatial genomics in understanding disease mechanisms, and diagnosing, and treating various conditions.	[**181**]
scRNA-seq, scATAC-seq	/	Human organoids	Constructing a human brain organoid development atlas to investigate the regulation of cell fate decisions.	[182]
scRNA-seq, scTCR-seq	Ovarian cancer (OC)	Human	A single-cell landscape of the OC ecosystem was depicted and revealed the heterogeneity of functional phenotypes and developmental origins of macrophages in tumor tissues and ascites.	[241]
Developmental biology				
Camallia-seq	/	Mouse	A new technology, Camallia-seq, enabled integrative analysis of chromatin accessibility, DNA methylation, transcriptome, as well as cell lineage information, bringing new insights into how cell fate decisions are regulated and how cell identities are maintained under different modalities.	[187]
scRNA-seq, spatial transcriptomics	/	Human and mouse	Precisely characterizing human embryonic limb development over time and space.	[194]
Pathways identification				
scRNA-seq, spatial transcriptomics, spatial proteomics	Cervical squamous cell carcinoma (CSCC)	Human	MP6 tumor cells interact with CAFs to shape an immune exclusionary microenvironment via the FABP5-mediated TGFβ pathway.	[195]
scRNA-seq, scATAC-seq	Neuroendocrine prostate cancer (NEPC)	Human and mouse	FOXA2 is a critical driver of the adeno-to-neuroendocrine lineage transition in prostate cancer that can activate the KIT pathway to maintain cancer cell proliferation.	[196]
scRNA-seq, snATAC-seq	Kidney Disease	Human and mouse	The Nfix-Ifi27 pathway can cause kidney fibrosis by promoting apoptosis.	[198]
Novel targets discovery				
scRNA-seq, snATAC-seq	Lymphangioleiomyomatosis (LAM)	Human and mouse	A HOX-PBX gene regulatory network controlled the survival of LAM cells.	[201]
scRNA-seq, spatial transcriptomics, spatial proteomics	Metastatic melanoma (MM)	Human	A TCF4-dependent regulatory network orchestrated multiple transcriptional programs leading to immunotherapy resistance.	[202]
scRNA-seq, spatial transcriptomics	Multiple primary lung cancers (MPLCs)	Human	TNFRSF18, associated with non-response to anti-PD-1 therapy was highly expressed in T&NK cells within MPLCs.	[203]
ICIs				
scRNA-seq, scTCR-seq, scBCR-seq	Epstein-Barr virus-associated gastric cancer (EBV + GC)	Human	Re-emerged clonotypes in ISG-15^+^CD8^+^ T cells after treatment among EBV (+) patients were detected and associated with effector T population expressing CXCL13 in responsive EBV (+) tumor, indicating their significant importance in tumor immunochemotherapy response.	[25]
scRNA-seq, TCR-seq	Localized bladder transitional cell carcinoma (TCC)	Human	Identifying distinct CD4^+^ and CD8^+^ T cell states within bladder tumors and highlighting the presence of cytotoxic CD4^+^ T cells and various CD8^+^ T cell subsets.	[206]
scRNA-seq, ACAT-seq	Hepatocellular carcinoma (HCC)	Human and mouse	TdLN-TTSM cells were primary memory T cells that respond to ICI treatment, representing the adoptive transfer of these cells as a promising immunotherapy approach.	[**207**]
ACT				
scRNA-seq, CITE-seq	B-cell Acute Lymphoblastic Leukemia (B-ALL)	Human	Lack of TH2 functionality might be the cause of relapse in CAR-T treatment.	[**209**]
TCR-seq, CITE-seq	Non-small cell lung cancer (NSCLC)	Human	A phenotypic signature based on CD39 and CXCL13 identified neoantigen-reactive T cells in fresh NSCLC.	[**210**]
Host-microbe interactions				
16 S rRNA sequencing, scRNA-seq	Intrahepatic cholangiocarcinoma (ICC)	Human	*P. fungorum* inhibited tumor growth through alanine, aspartate, and glutamate metabolism.	[**214**]
16 S rRNA sequencing, scRNA-seq	Gastric cancer (GC)	Mouse	*Streptococcus anginosus* promoted gastric tumorigenesis.	[**215**]
scRNA-seq, TCR-seq	Pan-cancer	Human and mouse	The commensal bacterium *Lactobacillus johnsonii* enhanced the efficacy of ICI therapy by modulating the stemness of CD8^+^ T cells.	[**216**]
Infectious diseases				
scRNA-seq, scBCR-seq	SARS-CoV-2	Human	Discovery and characterization of potent neutralizing antibodies from individuals with Omicron breakthrough infections.	[**145**]
scRNA-seq, BCR-seq, TCR-seq	SARS-CoV-2	Mouse	A systematical depiction of the immune landscape after vaccinating lipid nanoparticle-mRNA.	[**146**]
scRNA-seq, scCITE-seq, scTCR-seq, plasma proteomics, metabolomics	SARS-CoV-2	Human	Observing unique dynamics in the behavior of specific CD8^+^ T cells during the recuperation phase from COVID-19, among patients suffering from gastrointestinal sequelae.	[**219**]
Cardiovascular disease				
sc-RNA-seq, snRNA-seq, snATAC-seq, spatial transcriptomics	/	Human	Constructing the human cardiac landscape and paving the way for the anatomy and immunology of the heart.	[**227**]
scRNA-seq, scTCR-seq	Myocardial infarction (MI)	Human and mouse	TCR-M cells expressing Treg markers like Foxp3, Il2ra, and Ctla4 suppressed cardiac immune responses post-MI and improved cardiac function.	[**228**]
scRNA-seq, CITE-seq	Atherosclerosis (AS)	Human	Deciphering the immune landscapes in the plaques of AS and uncovering immune alterations related to clinical cardiovascular events.	[**230**]
Neurological and brain disease				
scRNA-Seq, proteomics	Schizophrenia	Human organoids	Identifying two disease-associated factors, BRN2 and PTN, which promoted neurogenesis, with PTN also inhibited apoptosis.	[**233**]
scRNA-seq, spatial transcriptomics	Ischemic stroke	Mouse	Two microglial subclusters, ischemic core-associated microglia (ICAM) and ischemic penumbra-associated microglia (IPAM) were identified in the brains of mice subjected to middle cerebral artery occlusion (MCAO).	[**24**]
scRNA-seq, spatial transcriptomics	Ischemic Stroke	Mouse	LGALS9-CD44 is a crucial pathway after ischemic injury. LGALS9 and CD44 exhibited opposite effects, in which upregulation of LGALS9 favored recovery from post-ischemic injury, whereas knockdown of CD44 diminished the therapeutic effect of LGALS9.	[**235**]

## The challenges of single-cell multi-omics

Single-cell multi-omics technologies, widely applied in both biological research and clinical practice, have been bolstered by advancements in experimental protocols and data analysis, as well as by a growing consensus on their significance. Despite these improvements, they still face hurdles that impede their widespread applications. These challenges and obstacles delineate the future development and trajectory of single-cell multi-omics. The following limitations and issues must be taken into account when performing single-cell multi-omics analyses.

Firstly, the high cost and strict sample requirements of single-cell technologies discourage many researchers [131]. Developing high-throughput, cost-effective, sample-friendly, convenient single-cell multi-omics technologies is a crucial issue, particularly for the application in clinical practice. The high cost of single-cell multi-omics restricts the measurement of large-scale cohorts, leading to data that is more often utilized for discovering new insights rather than for validation, which is commonly achieved by bulk RNA sequencing [25]. Another challenge is the strict requirement for sample quality. Fresh tissue samples are deemed appropriate for single-cell sequencing, whereas freeze-thaw samples are generally recommended for snRNA-seq [242, 243]. Besides, the quantity and viability of the cell suspension are also important factors in obtaining high-quality single-cell data and detecting all cell types in the tissue.

The emerging single-cell multi-omics broadens the multidimensionality beyond transcriptomes and raises more profound questions. Developing robust and advanced computational methods to integrate and analyze multi-dimensional single-cell data is a pressing challenge to be addressed for the maturation of single-cell multi-omics [7]. Importantly, these strategies need to consolidate the data across diverse dimensions and manage potentially significant differences in individual omics data, enhancing the understanding of cellular function and state. In some contexts, inconsistencies in information can occur among omics data, although the measurement is conducted simultaneously within the same cells [7, 129].

Key information missing or mismatch due to the technology is another issue. Sequencing depth directly affects the quality of the data obtained, and choosing the appropriate sequencing depth in scRNA-seq is crucial [244]. Deeper sequencing provides a more comprehensive gene expression profile, while insufficient sequencing depth may result in the loss of crucial information, impacting the annotation of cell types and the interpretation of their functions. Besides, some cellular heterogeneities may be not well presented in the transcriptome, and fail to define the state of the cell. High-throughput single-cell proteomics methods may work, which are not yet established [131]. Capturing the immune repertoire also faces inherent technical limitations. Instances such as measuring only TRA or TRB, or erroneous matching to more than two chains, do occur [25]. Addressing how to effectively handle these data requires more uniform rules. Besides, there are various causes for clonal expansion, including the pressure of tumor neoantigens or infectious diseases. Determining whether clonal expansion is due to a specific factor, like tumor antigen pressure, is a topic worth exploring. This may necessitate the creation of a dedicated immune repertoire database or the use of biochemistry methods to bidirectionally decode TCR and pMHC, among other approaches.

Doublet refers to two cells encapsulated into one reaction volume [245], which may confound downstream analysis, including atlas construction, DEG analysis, and cell trajectory inference. The emergence of the doublet is especially evident when a large amount of cell volume is put in. Developing a suitable means of assessing the removal of double cells can prevent many strange problems from arising [245, 246].

Altogether, overcoming these challenges to integrate high-quality, multimodal single-cell multi-omics data is essential. Understanding the patterns of normal tissue function and disease progression from a single-cell perspective is inseparable from these problems being solved.

## Conclusions

Single-cell sequencing provides unprecedented resolution to identify multicellular connectivity and heterogeneity. Multi-omics technologies have empowered the scRNA-seq to break through the original limitations associated with relying solely on transcriptome gene expression profiles. Integrated with transcriptome, genome, metabolome, proteome, TCR/BCR, epigenome, etc., and broadening the axes of timescale and spatial information, multidimensional information provides a comprehensive snapshot of cell types and states. In the present review, we discuss the established cutting-edge single-cell multi-omics technologies over the past decades. Additionally, burgeoning computational biology technologies are another major step toward uncovering and deciphering the secrets within the multidimensional datasets. These bioinformatics tools link the datasets from different modalities elucidate the function within different cell types, and provide a wide range of information through mathematical modeling and artificial intelligence methods. From the view of clinical practice, we highlight the applications in tumor immunology, therapeutic technologies, and drug treatments. Especially for the discovery of new cell populations and new targets, as well as evaluation and interpretation of drugs and therapeutics, such as PD1 and CAR-T, are elucidated.

Collectively, single-cell multi-omics methods have essentially expanded the tools to discover the rich resources and understand the inner workings of biological processes at the single-cell level. Given that future multi-omics studies will aid in addressing numerous biological research and clinical practices, the technologies will become the standard toolkit for studies on molecular cell biology.

## Data Availability

No datasets were generated or analysed during the current study.

## Abbreviations

scRNA-seq: Single-cell RNA sequencing
scTCR-seq: Single-cell T cell receptor sequencing
scBCR-seq: Single-cell B cell receptor sequencing
scATAC-seq: Single-cell assay for transposase-accessible chromatin using sequencing
TFs: Transcription factors
PCA: Principal component analysis
UMAP: Uniform manifold approximation and projection
t-SNE: t-distributed stochastic neighbor embedding
DEGs: Differentially expressed genes
GO: Gene ontology
KEGG: Kyoto encyclopedia of genes and genomes
GSVA: Gene set variation analysis
SCENIC: Single-cell regulatory network inference and clustering
CCA: Canonical correlation analysis
MMN: Mutual nearest neighbors
4sU: 4-thiouridine
U: Uracil
C: Cytosine
A: Adenine
G: Guanine
scSLAM-seq: Single-cell, thiol-(SH)-linked alkylation of RNA for metabolic labelling sequencing
CMV: Cytomegalovirus
UMI: Unique molecular identifier
EU: 5-ethynyl-uridine
EdU: 5-Ethynyl-2-deoxyuridine
EE: Enteroendocrine
gRNAs: Guide RNAs
TME: Tumor microenvironment
ISH: In situ hybridization
ISS: In situ sequencing
smFISH: Single-molecule fluorescence ISH
seqFISH: Sequential FISH
MERFISH: Multiplexed error-robust FISH
RCA: Rolling-circle amplification
RCP: Rolling-circle product
FISSEQ: Fluorescent in situ sequencing
STARmap: Spatially-resolved transcript amplicon readout mapping
3D: Three-dimensional
CNS: Central nervous system
ROI: Regions of interest
LCM: Laser capture microdissection
DSP: GeoMx digital spatial profiler
Geo-seq: Geographical position sequencing
PC: Photocleavable
ICI: Immune checkpoint inhibitor
NSCLC: Non-small cell lung cancer
NGS: Next-generation sequencing
ST: Spatial transcriptomics
HDST: High-definition spatial transcriptomics
Stereo-seq: Spatial enhanced resolution omics-sequencing
Decoder-seq: Dendrimeric DNA coordinate barcoding design for spatial RNA sequencing
DBiT-seq: Deterministic barcoding in tissue for spatial omics sequencing
spatial CUT&Tag-RNA-seq: Spatial assay of cleavage under targets and tagmentation and RNA sequencing
spatial CITE-seq: Spatial co-indexing of transcriptomes and epitopes for multi-omics mapping by highly parallel sequencing
scWGS: Single-cell whole-genome sequencing
SNP: Single nucleotide polymorphism
eQTL: Expression quantitative trait loci
G&T-seq: Genome and transcriptome sequencing
DR-seq: gDNA-mRNA sequencing
5mC: 5-methylcytosine
scRRBS: Single-cell reduced representation bisulfite sequencing
scWGBS: Single-cell whole-genome bisulfite sequencing
scM&T-seq: Single-cell methylome and transcriptome sequencing
scTrio-seq: Single-cell triple omics sequencing
ChIP-seq: Chromatin immunoprecipitation sequencing
scChIP-seq: Single-cell ChIP-seq
scCUT&Tag: Single-cell CUT&Tag
CoTECH: Combined assay of transcriptome and enriched chromatin binding
scMS: Mass spectrometry-based single-cell proteomics
CITE-seq: Cellular indexing of transcriptomes and epitopes by sequencing
Treg: T regulatory cell
SUGAR-seq: SUrface-protein Glycan And RNA-seq
TILs: Tumor-infiltrating lymphocytes
LacNAc: N-acetyllactosamine
IR: Immune repertoire
pMHC: Peptide-loaded major histocompatibility complex
V: Variable
D: Diversity
J: Joining
C: Constant
CDR3: Complementarity-determining region 3
STARTRAC: Single T cell analysis by RNA sequencing and TCR tracking
LIBRA-seq: Linking B-cell Receptor to Antigen specificity through sequencing
BarBIQ: Barcoding bacteria for identification and quantification
microSPLiT: Microbial split-pool ligation transcriptomics
INVADEseq: Invasion-adhesion-directed expression sequencing
SAHMI: Single-cell Analysis of Host-Microbiome Interactions
MS: Mass spectrometry
NMR: Nuclear magnetic resonance
CCIs: Cell-cell interactions
FT: Fucosyltransferase
DC: Dendritic cell
ReDeeM: Regulatory multi-omics with Deep Mitochondrial mutation profiling
CSCC: Cervical squamous cell carcinoma
MP: Meta-program
CAF: Cancer-associated fibroblast
TGFβ: Transforming growth factor β
NEPC: Neuroendocrine prostate cancer
snATAC-seq: Single-nucleus ATAC sequencing
LAM: Lymphangioleiomyomatosis
MPLCs: Multiple primary lung cancers
ACT: Adoptive cell therapy
EBV: Epstein‒Barr virus
TCR-T: T cell receptor-T cell
CAR-T: Chimeric antigens receptor-T cell
PDX: Patient-derived xenograft
ALL: Acute lymphoblastic leukemia
nAb: Neutralizing antibody
MI: Myocardial infarction
snRNA-seq: Single-nucleus RNA sequencing
AS: Atherosclerosis
AD: Alzheimer’s disease
